# “Watch Me Grow- Electronic (WMG-E)” surveillance approach to identify and address child development, parental mental health, and psychosocial needs: study protocol

**DOI:** 10.1186/s12913-021-07243-0

**Published:** 2021-11-17

**Authors:** V. Eapen, S. Woolfenden, V. Schmied, B. Jalaludin, K. Lawson, S. T. Liaw, R. Lingam, A. Page, S. Cibralic, T. Winata, A. Mendoza Diaz, C. Lam-Cassettari, J. Burley, K. Boydell, P. Lin, A. Masi, I. Katz, A. Dadich, J. Preddy, J. Bruce, S. Raman, J. Kohlhoff, J. Descallar, L. Karlov, C. Kaplun, A. Arora, B. Di Mento, M. Smead, K. Doyle, R. Grace, T. McClean, V. Blight, A. Wood, K. Hazell Raine

**Affiliations:** 1 School of Psychiatry, Faculty of Medicine, University of New South Wales, Sydney, Australia; 2 South Western Sydney Local Health District, Liverpool, Australia; 3 Sydney Children’s Hospital Randwick, Randwick, Australia; 4 School of Nursing and Midwifery, Western Sydney University, Sydney, Australia; 5 Ingham Institute for Applied Medical Research, Liverpool, Australia; 6 School of Business, Western Sydney University, Sydney, Australia; 7 Population Child Health Research Group, School of Women’s and Children’s Health, Faculty of Medicine, University of New South Wales, Sydney, Australia; 8 WHO Collaborating Centre for eHealth, University of New South Wales, Sydney, Australia; 9 School of Medicine, Western Sydney University, Campbelltown, Australia; 10 Black Dog Institute, Sydney, Australia; 11 Social Policy Research Centre, Faculty of Arts, Design, & Architecture, University of New South Wales, Sydney, Australia; 12 Murrumbidgee Local Health District, Wagga Wagga, Australia; 13 Karitane, Carramar, Australia; 14 TeEACH –Transforming early Education and Child Health Research Centre, Western Sydney University, Sydney, Australia; 15 School of Health Sciences, Western Sydney University, Sydney, Australia; 16 Sydney Local Health District, Camperdown, Australia; 17 Uniting, Sydney, Australia

**Keywords:** Child development, Parent mental health, Psychosocial needs, Screening, Developmental surveillance, Service navigation, Virtual care, Digital solution, Online health service, Electronic platform

## Abstract

**Background:**

The COVID-19 pandemic and the associated economic recession has increased parental psychosocial stress and mental health challenges. This has adversely impacted child development and wellbeing, particularly for children from priority populations (culturally and linguistically diverse (CALD) and rural/regional communities) who are at an already increased risk of health inequality. The increased mental health and psychosocial needs were compounded by the closure of in-person preventive and health promotion programs resulting in health organisations embracing technology and online services. Watch Me Grow- Electronic (WMG-E) – developmental surveillance platform- exemplifies one such service.

WMG-E was developed to monitor child development and guide parents towards more detailed assessments when risk is identified. This Randomised Controlled Trial (RCT) aims to expand WMG-E as a digital navigation tool by also incorporating parents’ mental health and psychosocial needs. Children and families needing additional assessments and supports will be electronically directed to relevant resources in the ‘care-as-usual’ group. In contrast, the intervention group will receive continuity of care, with additional in-person assessment and ‘warm hand over’ by a ‘service navigator’ to ensure their needs are met.

**Methods:**

Using an RCT we will determine: (1) parental engagement with developmental surveillance; (2) access to services for those with mental health and social care needs; and (3) uptake of service recommendations. Three hundred parents/carers of children aged 6 months to 3 years (recruited from a culturally diverse, or rural/regional site) will be randomly allocated to the ‘care-as-usual’ or ‘intervention’ group. A mixed methods implementation evaluation will be completed, with semi-structured interviews to ascertain the acceptability, feasibility and impact of the WMG-E platform and service navigator.

**Conclusions:**

Using WMG-E is expected to: normalise and de-stigmatise mental health and psychosocial screening; increase parental engagement and service use; and result in the early identification and management of child developmental needs, parental mental health, and family psychosocial needs. If effective, digital solutions such as WMG-E to engage and empower parents alongside a service navigator for vulnerable families needing additional support, will have significant practice and policy implications in the pandemic/post pandemic period.

**Trial registration:**

The trial (Protocol No. 1.0, Version 3.1) was registered with ANZCTR (registration number: ACTRN12621000766819) on July 21st, 2021 and reporting of the trial results will be according to recommendations in the CONSORT Statement.

## Background

Due to the COVID-19 pandemic, families, particularly those from disadvantaged backgrounds experienced significant psychosocial stress and mental health challenges [1]. In Australia, for example, despite the low infection rates and consequent adverse social and economic impact, approximately 1.6 million families experienced financial hardship due to COVID-19 associated job losses and financial stress [2], resulting in an extra 780,000 Australian children living in families experiencing employment stress [3, 4]. Further, parental mental health difficulties consequent to the pandemic can adversely impact children’s development and wellbeing [3, 5, 6]. Specifically, families from priority populations such as culturally and linguistically diverse (CALD) as well as from regional and rural communities (i.e., areas that lie beyond the major cities) are particularly likely to experience health care inequality [7, 8]. These risks were intensified by the closure of in-person preventative and health promotion programs, such as child wellbeing checks. This combination of circumstances, substantially reduced access to resources for vulnerable families during the pandemic [9].

When in-person consultations closed, health organizations embraced technology and offered online services to continue providing access to services [10]. Watch Me Grow – Electronic Platform (WMG-E) is an example of one such innovative platform that can help health services reach vulnerable families in their homes, in the community, or during opportunistic contacts (e.g. immunisation) with health professionals such as General Practitioners (GPs) and Child and Family Health Nurses (CFHN) [9]. WMG-E is a web-based application that incorporates the Centre for Disease Control (CDC) “Learn the Signs Act Early (LTSAE)” program which consists of age-appropriate developmental checklists and ‘red flags’ for parental concern of children from birth to 5 years (see measures section for a detailed description of the LTSAE) [11]. In Australia, the New South Wales (NSW) State Government has incorporated the LTSAE program into the *My Personal Health Record Blue Book* developmental surveillance program [12]. Furthermore, translations of the LTSAE are available for the 19 of the most commonly spoken languages in NSW [12]. WMG-E serves as a digital tool to engage and empower parents to actively participate in their children’s developmental monitoring. Parents are provided with anticipatory guidance and automatic reminder emails at the recommended ages and stages for child developmental checks for ongoing developmental monitoring. When parents raise any concerns on the LTSAE red flag items, they are guided to seek more comprehensive assessments by health service providers.

The proposed study will expand WMG-E by incorporating screeners for parents’ mental health and unmet psychosocial needs (e.g., financial support, housing) to optimise families’ wellbeing. Like the developmental screener, when mental health and/or unmet psychosocial risks are indicated, families will be directed to relevant resources electronically through WMG-E in the control group while the intervention group will be guided to receive more detailed assessments and provided ongoing care to ensure the uptake of recommended services. This study also aims to evaluate whether additional support through a ‘service navigator’ (i.e., WMG-E + service navigator) increases family access and uptake of services for families identified as at high-risk by WMG-E. Previous research conducted by Hughes and colleagues [13] has shown that the involvement of a service navigator can enhance families’ connections to services. The role of the service navigator, similar to that of the care coordinator in Hughes et al.’s [13] study, will be to review the parents’ mental health, psychosocial, and developmental screener results, connect them with relevant support services as needed, facilitate access and uptake of recommended services and provide continuity of care to ensure that their needs are met. Specifically, in the intervention group, the navigator will contact the family initially to discuss and clarify the concerns that were raised by the family while completing WMG-E. Once the service navigator has clarified the family’s needs, they will provide guidance to the family on available services and supports in their local area, whom to speak to at relevant agencies, specific questions to ask, and, if necessary, help complete referral forms and where needed directly link them with the service. The service navigator will then contact the family within a 2-week period to determine whether they were able to successfully engage with the available services and supports. The service navigator will continue to contact the family on a biweekly basis to check in regarding their engagement with recommended services and determine whether they have any additional concerns or unmet needs.

A “*virtual care approach*” [14] will be used to focus predominantly on families from priority populations from CALD and rural/regional backgrounds, given the higher risk for health care inequality [7, 8]. As approximately 3.6 billion people use social media, the use of web-based applications has become ubiquitous, including those from priority populations [15]. Therefore, by providing a web-based application which is accessible, affordable, and easy to use we hope to improve access and uptake of screening for mental health, psychosocial needs, and developmental needs in some of the most vulnerable populations in NSW. As for newly arrived immigrants with young children, community linkages with friends, family, and GPs are pivotal [16], we will utilise GPs, play groups, social care agencies, government and non-governmental organisations (NGOs) to engage parents, normalise and de-stigmatise mental health and psychosocial screening, and provide urgent targeted interventions.

## Objectives

The primary objectives of the Randomised Controlled Trial (RCT) component involving families of children aged 6 months to 3 years from CALD, regional and rural communities in NSW are to evaluate: (1) the engagement with developmental surveillance; (2) access to services for those with mental health and social care needs; and (3) uptake of service recommendations. Secondary objectives will include examining (1) the effectiveness of the WMG-E platform in identifying and addressing parental mental health, parental psychosocial and child developmental needs; and (2) whether a service navigator, in addition to the WMG-E platform, increases family wellbeing by facilitating access to relevant services and by providing continuity of care.

The objective of the implementation evaluation is to ascertain the acceptability, feasibility and the impact of the WMG-E platform and service navigator.

### Randomised controlled trial

Using an RCT methodology we will compare a ‘care-as-usual’ (CaU) group with an intervention group. Both groups will complete parental mental health, psychosocial needs, and child developmental screeners via WMG-E. Furthermore, both groups, if risk is identified, will receive electronic resources guiding them to health services that can be accessed for more detailed assessments.

The intervention group will be provided with ongoing monitoring, via a service navigator, to ensure uptake of recommendations and access to additional support for those identified to be at risk. The service navigator will first triage intervention families based on their level of risk as indicated by the WMG-E platform which screens for developmental, parental mental health, and psychosocial risk. Participants who indicate no developmental concerns on the LTSAE and psychosocial concerns on the WE CARE questionnaire [17] and have scores lower than 20 on the Kessler Psychological Distress (K10) scale [18] will be considered low risk. Participants who indicate one or more developmental and/or psychosocial concerns and/or have scores ranging from 20 to 24 on the K10 will be considered medium risk. Participants who have scores equal to or greater than 25 on the K10 and/or indicate risks of homelessness, domestic violence, loss of electricity, or food insecurity will be considered high risk. Completing the screening tools on the WMG-E platform will take between 10 and 15 min.

Families with lower level risks will be connected with local services and might also be provided with information on online resources (e.g., free to access online parenting programs such as ParentWorks [19] or Movember Family Man [20]) or therapist-assisted interventions (e.g., online or in-person Parent-Child Interaction Therapy [21]). For families who are at high risk, in addition to receiving resources and linkages to services as mentioned above, they will receive in-person assessments (through a KIDS-Connect hub [22] with capacity to reach families at homes or other community facilities) to determine the complexity of health needs followed by appropriate referrals and a ‘warm hand-over’ (i.e., directly linking patients to services in a supportive manner) to recommended services. The duration of in-person (or digital as indicated in extreme circumstances) assessments will depend on the needs identified by the WMG-E, however, these assessments generally range from 30 to 60 min [22]. Once the in-person assessment is completed and families are linked with recommended services, the type and duration of services they receive will depend on their needs. For example, families with psychological needs may receive ten 60-min sessions with a psychologist while families with unmet psychosocial needs who access services such as Uniting (community service agency) [22, 23] may receive 2–3 sessions targeted at addressing psychosocial needs. Furthermore, these families will receive follow-up support by the navigator to ensure engagement with recommended services and continuity of care. Follow-ups will occur fortnightly via telephone for a duration of 10 to 20 min.

We hypothesise that in the first 6 months, the intervention group will have better access and engagement with services due to this group not only getting linked up with services but also continuity of care through the service navigator to ensure the uptake of recommended services. Further we hypothesize that, families in the intervention group that are connected with services will also show a significantly greater increase in wellbeing relative to the control group, at the 12 month follow up. This is because, via the service navigator, they will have assistance in connecting with services and ongoing monitoring and support to ensure that their needs are being met.

### Implementation evaluation

The implementation evaluation will involve semi-structured interviews conducted with participants and stakeholders (e.g., service providers, policy makers) to clarify their experiences with the WMG-E platform and the service navigator, and to identify facilitators and barriers to intervention success. Specifically, the interviews will consider experiences with using the WMG-E app and the service navigator, perceived feasibility and benefits, ways to improve the WMG-E platform and user experience, and differences between routine clinic-based health services and community-based approaches to services.

## Methods

### Phase 1: randomised controlled trial

#### Design

The study design is a two (group: intervention versus control) by three (time: baseline [Time 1], post-assessment [Time 2], and follow-up [Time 3]) RCT.

#### Participants

Participants will include 300 parents/caregivers (150 from Fairfield Local Government Area (LGA) and 150 from Murrumbidgee Local Health District (LHD)) of children aged 6 months to 3 years (inclusive).

#### Setting

Fairfield (population: 198,817; 102 km^2^) [24] is an urban area and has a high proportion of CALD families, with about 70% of residents speaking a language other than English at home [24]. Murrumbidgee LHD (population: 77,652; 125,242 km^2^) [24] is considered mostly regional with one remote area making access to health care a challenge for most families with young children.

#### Procedure

##### Recruitment

Families attending child and family health services, refugee health services, supported playgroups, parenting groups, NGO services, GP clinics, special paediatric clinics, or Child and Family Health Nurse services in Murrumbidgee LHD and Fairfield LGA will be informed about the study by their service provider. They will also be provided with a quick response (QR) code with a hyperlink to the WMG-E app which they can access using tablet devices provided by the service they are attending or using a personal smartphone. Families will be excluded from the research if they are allocated to the control group but are in “acute crisis” based on their questionnaire responses (i.e., K10 > 25 and/or risk of homelessness, domestic violence, loss of electricity, or no food). These families will automatically be considered RCT dropouts and will be contacted by a researcher who will assist them in engaging with services.

##### Randomisation

Upon completion of the consent forms and Time 1 questionnaires, families will be randomised to either the intervention or CaU group. Computerized randomisation software will be used to create a randomisation table via REDCap (a secure web application for building and managing online surveys and databases) [25]. Participants will be assigned randomly to each arm ensuring a 50:50 ratio.

##### Assessments

**Fig. 1:**
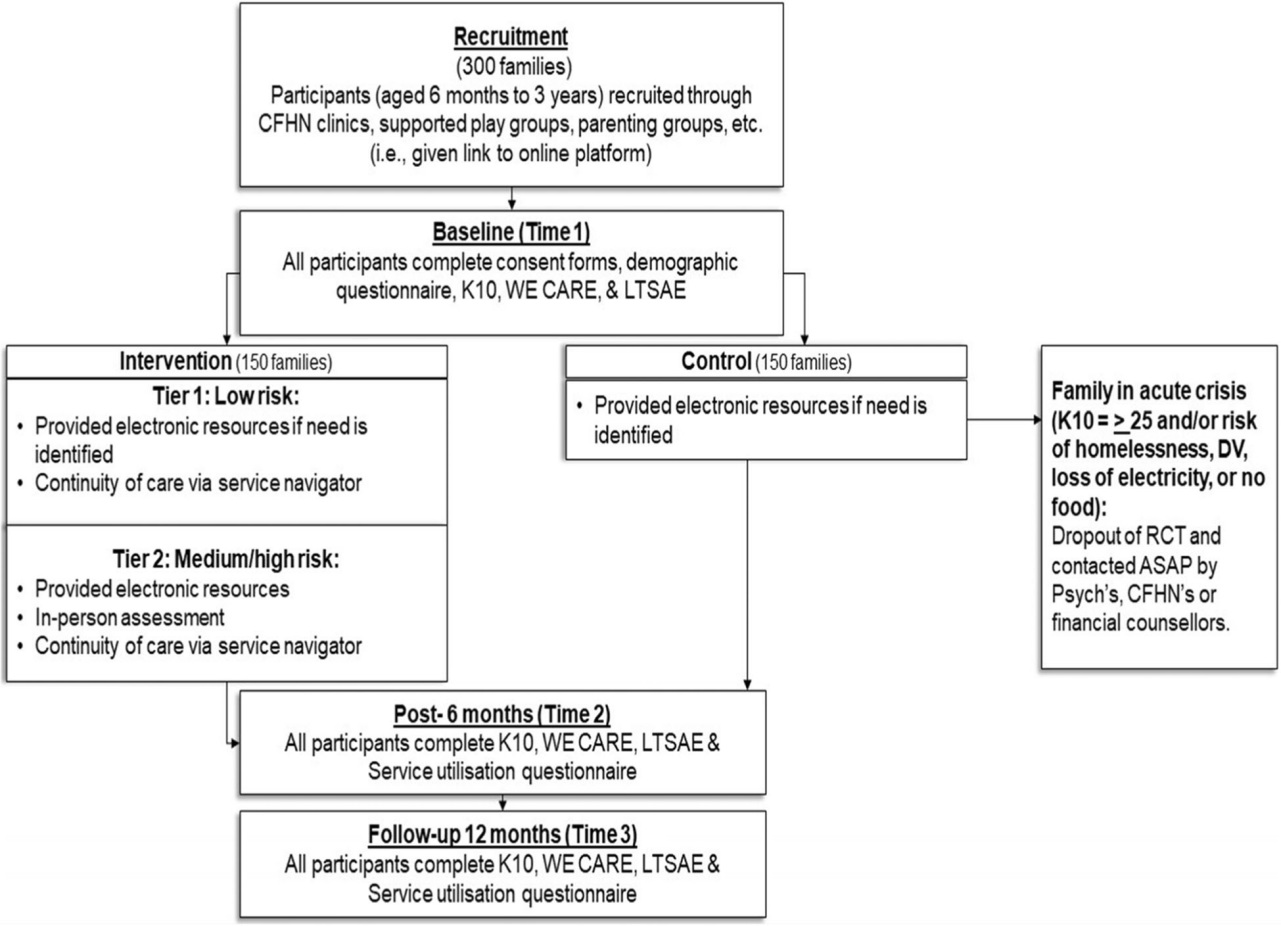
Participant flow through the study. Note: K10 = Kessler Psychological Distress; LTSAE = Learn the Signs Act Early

**Fig. 2:**
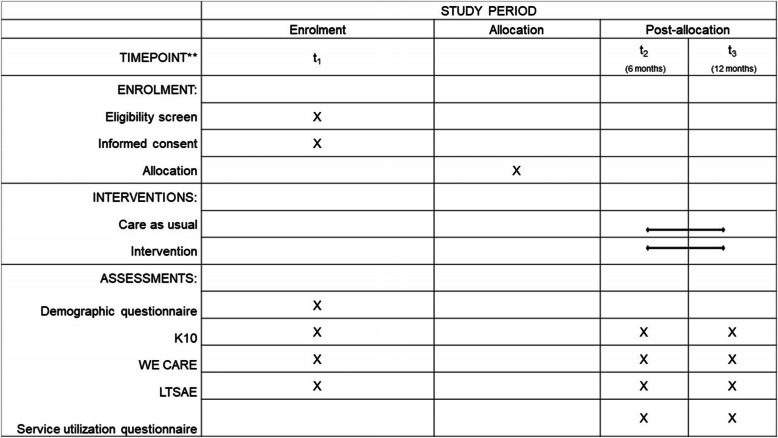
SPIRIT flow diagram of the WMG-E study. Note: K10 = Kessler Psychological Distress; LTSAE = Learn the Signs Act Early

All participants will complete consent forms and a demographic information questionnaire along with the study questionnaires through the REDCap platform. These questionnaires will be made available in English, Vietnamese, Arabic, and simplified Chinese (the four most common languages spoken in NSW). Where translated versions of questionnaires exist, they will be used, otherwise a licensed interpreter will be commissioned to translate the questionnaires during assessment. Those who cannot speak English will be linked up with services with access to interpreters as part of their service delivery. The demographic questionnaire, which will be completed at trial entry (baseline), will include the child’s date of birth, the child’s gender, whether the child was born premature, the child’s country of birth, the mother’s age, the mothers education level, the mother’s country of birth, the fathers age, fathers education level, the father’s country of birth, parent marital status, socioeconomic status, and the main language spoken in the home. The study questionnaires will be completed at three time points: baseline [Time 1], six-months post-intervention [Time 2], and 12-months post-intervention [Time 3]. See Figs. 1 and 2 for a summary of participant flow through the study.

#### Groups

##### Intervention group

Participants allocated to the intervention group whose responses to the Time 1 questionnaires indicate mental health, psychosocial, and/or developmental risks will receive an immediate message on the device that they used to complete the questionnaires. The message will suggest that they might require additional assessment/support and will recommend that they seek relevant local services. In the intervention group, the participant’s results will be emailed to a service navigator who will then contact participants via telephone and direct them to relevant care pathways depending on their identified needs. This might include, for example, further assessments and/or referral pathways as deemed appropriate. The service navigator will initially attempt to contact participants four times (two telephone calls and two emails, contact attempts will be tracked on an excel spreadsheet). If participants do not engage with the service navigator they will be considered as drop-outs from the RCT.

##### Care-as-usual group

Participants allocated to the CaU will complete Time 1 questionnaires and, if indicated, will also receive a message that their responses to the questionnaires suggest developmental, psychological, and/or psychosocial risk and to seek assistance from local services/organisations. They will then be invited via email to complete Time 2 and Time 3 questionnaires. The control group will not have a service navigator and will not receive continuity of care and support.

#### Measures

The following measures have been incorporated into the WMG-E weblink to identify mental health, psychosocial, and development risks.

##### Parent mental Health

Parent psychological distress will be assessed using the Kessler Psychological Distress (K10) scale [18] – a ten-item measure. Using a five-point Likert scale, parents are required to indicate how often they have experienced symptoms of depression and anxiety as well as somatic symptoms over the past 4 weeks. One indicates ‘none of the time’ and five, ‘all of the time’. Each item score is combined to calculate the total score. Scores of 20 to 24 (inclusive) indicate mild psychological distress, 25 to 29 (inclusive) indicate moderate psychological distress, and 30 to 50 (inclusive) indicate severe psychological distress. The K-10 has good validity and reliability in various settings and populations [26–28].

##### Parent psychosocial needs

Parent psychosocial needs will be assessed using the WE CARE questionnaire [17]. The questionnaire consists of six (yes/no) items querying psychosocial needs: childcare, employment, homelessness, food security, education, and utilities. The measure also includes follow-up questions that appear if participants indicate “yes” to any of the six items. These items will be summed up to create a score out of 6. These follow-up questionnaires require participants to answer on a three-point scale (yes, no, or maybe later), whether they require additional support for addressing their unmet psychosocial need/s. The measure has good psychometric properties, including excellent test-retest reliability (r. = 0.92) [17].

##### Developmental risk

Developmental risk will be assessed using the appropriate Learn the Signs Act Early (LTSAE) questionnaire for their age [11]. The LTSAE is a short developmental surveillance questionnaire for children aged 2 months to 5 years that includes eight ‘red flag’ questions concerning the development of social and emotional, language/communication, cognitive, and movement/physical. The caregiver is asked to indicate whether their child engages in each of the developmental behaviours by indicating “yes”, “no” or “not sure”. The items will be summed up to create a score out of 8.

##### Service utilisation

As a way to track participants service utilisation, participants will be asked to complete a questionnaire, developed by the researchers, about the services that they are accessing. The following questions are included in the questionnaire: Do you or your child currently attend any health services? (question 1); if participants indicate ‘Yes’ on question 1, they will also be asked: Do you and/or your child currently attend any of the following services? (Psychologist, Social worker, Paediatrician, Occupational therapist, Speech pathologist, Early childhood education/intervention centres, Other) (question 2); who is receiving this service (you, your child, both)? (question 3); when did you/your child begin attending the service/s? (question 4); and how frequently do you/your child attend the service (e.g., weekly, monthly)? (question 5).

#### Analysis

##### Sample size calculations

A sample of 244 families will provide greater than 99% power in finding an increase from the current service utilisation of 30% doubling it to 60% in the intervention group with statistical significance at 5%. Taking non- and partial-adherence and sample dropout into account at 5%, the required sample size was revised to 300 (150 per group)*.*

##### Statistical analysis

A multilevel models estimated by restricted maximum likelihood (REML) with logit link function will be used to model service utilisation over the 3 time points. The main predictors of interest will be time, group and a time by group interaction which will compare service utilization between the groups at each time point. The analysis will be adjusted for the caregivers, socioeconomic status, and the main language spoken in the home.

A multilevel model estimated by restricted maximum likelihood (REML) with identity link function will be used to model the K10, WE CARE and LTSAE totals. The main predictors of interest are time, group and time by group interaction. The analysis will be adjusted for the caregivers, socioeconomic status, and the main language spoken in the home.

##### Economic analysis

Economic evaluation will assess the cost effectiveness (cost utility) of the intervention against usual care. Costs will include the care coordinator for the intervention group and the costs of completed service referrals for both trial arm. Effectiveness will be measured by converting K-10 scores into utilities using a mapping algorithm that converts scores to EQ-5D values [29]. REML will also be applied to assess significant changes in costs and outcomes. An Incremental Cost Effectiveness Ratio (ICER) will then be generated with values $42,000–$67,000 considered to be cost effective [30]. Implementation costs from roll-out will be estimated, including the eligible population size, the number of service coordinators required and service referrals. Sensitivity analysis will then vary key parameters such as service uptake and a threshold analysis will estimate the minimum service engagement required to maintain cost effectiveness in implementation. A value of information analysis will estimate statistical uncertainty and the potential value from further research to optimize implementation.

### Phase 2: implementation evaluation

#### Design

Semi-structured one-on-one qualitative interviews lasting between 30 to 60 min will be conducted with participants and stakeholders via telephone or video call. Participants not proficient in English will have access to interpreters.

#### Participants

Participants will include approximately 30 families who participated in the study (15 from the CaU group and 15 from the intervention group) and 30 key stakeholders (e.g., CFHN, service providers, policy makers).

#### Recruitment

Participants in the wider RCT will be asked to indicate their consent on the RCT consent form to being contacted regarding participation in the qualitative component of the study. Those who indicate ‘yes’, will be contacted directly by a researcher after the RCT 12-month follow-up. Furthermore, the study will be advertised via flyers posted at participating sites in both Fairfield LGA and Murrumbidgee LHD and all RCT participants and stakeholders will be emailed the flyer.

#### Data collection and analysis

Participants who have indicated that they wish to take part in the interview will be contacted via email by a researcher to arrange an interview date and time. Once arranged, the researcher will email participants an information sheet and consent form. The research officer will then contact participants via telephone or video call at the scheduled time. Prior to the interview, the research officer will review the information sheet with the participant, ensuring that the consent form has been signed and returned.

The semi-structured interviews will be theoretically framed using the Consolidated Framework for Implementation Research [CFIR] and will consider barriers and facilitators for effective rollout. In addition, we will assess key implementation metrics including acceptability, adoption, appropriateness, fidelity, coverage, and sustainability [31–33]. Interviews will be digitally recorded, transcribed verbatim, and thematically analysed with an iterative framework analysis using NVivo Pro 12 software.

### Data storage and record retention

All participant data collected through REDCap will be stored securely on University of New South Wales REDCap servers which are password protected. All interview recordings and operational trial data (e.g., hard copies of flyers, ethics documentation) will be stored electronically on the Southwest Sydney Local Health District password-protected network drives which are accessible to a small number of Chief Investigators. No external personnel will have access to the data. The electronic data and paper files will be kept for 25 years, and the database will be deleted at the end of this time.

### Management of the project/governance

**Fig. 3:**
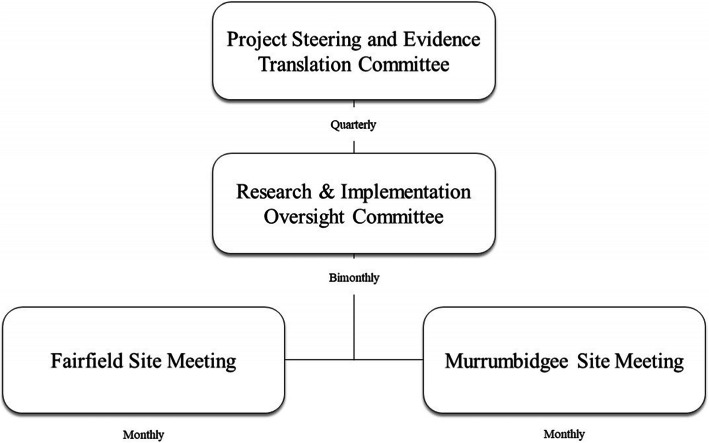
WMG-E Clinical Governance Framework

The clinical governance of the project (shown in Fig. 3) will be established through a top-down level framework. Firstly, the Project Steering and Evidence Translation Committee (highest-level) will be overseeing the Child and Family Health Services at the state level of NSW. This steering committee will include chief investigators, senior executives, and clinical stream leaders from each site (Fairfield and Murrumbidgee), partner organisations and consumer representatives, and will convene quarterly to oversee and guide the study. Secondly, a research and implementation committee, comprised of all academic researchers, clinicians, and project staff, will support the Project Steering and Evidence Translation Committee and will be convened bi-monthly to manage study execution. Research site groups (Fairfield and Murrumbidgee) will be responsible for the daily operation of the study, including collecting, assessing, reporting, and managing solicited and spontaneously reported adverse effects. As this study does not involve any investigational therapeutic goods or devices, all adverse events and other unintended effects of trial intervention or conduct will be reported to the relevant authorities as per the principles of the NHMRC National Statement on Ethical Conduct in Human Research guidelines [34]. This information will also be reported to the Data Monitoring Committee (DMC) and included as part of the project’s annual progress report to the relevant ethics committee.

An independent DMC comprising of experts in clinical trial conduct in health services research, statistics, mental health, and child development will meet once every 6 months to monitor the quality of the trial data and safety of research participants. DMC will monitor blinded response variables and safety outcomes for early dramatic benefits or potential adverse events and provide reports to the investigators on recommendations to continue or temporarily halt recruitment to the study. The committee will be governed by a charter that will outline their responsibilities, procedures, and confidentiality.

### Auditing

The study may be audited by the South Western Sydney LHD ethics committee and inspected by government regulatory authorities. Any source information and other study files will be made accessible at all study sites (SWSLHD and MLHD) at the time of auditing and inspection during the course of the study and after the completion of the study.

## Discussion

Families from disadvantaged backgrounds face a fragmented health system with financial, social, and structural barriers. Even when services are available, engagement and access to services are compounded by cultural and linguistic barriers as well as geographic challenges. These are exacerbated by the pandemic and the consequent impact on in-person services.

This study will address these challenges by testing the WMG-E web-app as a digital navigation tool to engage parents at home or through opportunistic contacts with health, early childhood education or other community/social care services. Extending earlier research using WMG-E to engage parents in developmental monitoring [35], this study will include parental mental health and psychosocial needs, alongside child developmental needs. Thus, this study will test the acceptability, effectiveness, and cost-effectiveness of WMG-E for families of children aged 6 months to 3 years (inclusive) in a predominantly CALD community and a regional/rural community to increase engagement of families with services and better identification of their needs.

Furthermore, this study will specifically evaluate whether a service navigator will increase family wellbeing by facilitating access to relevant services. Families in the intervention group are expected to experience significantly improved parental mental health, psychosocial wellbeing, and development outcomes compared to the control group. By targeting CALD and rural/regional communities, we will demonstrate the feasibility of the WMG-E for the most vulnerable and disadvantaged families. Following this, the online platform will be ready for national implementation with potential for international adaptation.

### Impact on policy and practice

If findings provide evidence that engaging parents in mental health, psychosocial, and developmental screening via an electronic platform improves parental engagement and access to services, the study will have significant practice and policy implications for supporting vulnerable families. Moreover, the study will serve to clarify the factors that help and hinder the implementation and sustainability of a digital tool within routine services using opportunistic contacts with families. Specifically, by exploring the five domains of the CFIR (intervention characteristics, inner/outer settings, characteristics of individuals involved in implementation and process [36, 37]), we will ascertain the acceptability and cultural appropriateness of the tool, as well as barriers that hinder adoption. This will generate the evidence needed to evaluate the factors influencing program implementation at scale.

### Limitations

Due to the nature of the intervention, it is not possible for participants or service navigators to be blinded to study conditions. Furthermore, the services that participants are connected with will vary depending on their needs. It will therefore be difficult to determine whether improved outcomes are due solely to the service navigator, the service attended, or a combination of both.

## Data Availability

Not applicable.

## Abbreviations

CALD: Culturally and linguistically diverse
CaU: Care-as-usual
CDC: Centre for Disease Control
CFHN: Child and Family Health Nurses
GPs: General Practitioners
ICER: Incremental Cost Effectiveness Ratio
K10: Kessler Psychological Distress
LGA: Local Government Area
LHD: Local Health District
LTSAE: Learn the Signs Act Early
NGOs: Non-governmental organisations
NSW: New South Wales
QR: Quick response
RCT: Randomised Controlled Trial
RELM: Restricted maximum likelihood
WMG-E: Watch Me Grow – Electronic Platform
